# Ancestral role of *Pax2/5/8* in molluscan brain and multimodal sensory system development

**DOI:** 10.1186/s12862-015-0505-z

**Published:** 2015-10-28

**Authors:** Tim Wollesen, Sonia Victoria Rodríguez Monje, Christiane Todt, Bernard M. Degnan, Andreas Wanninger

**Affiliations:** Department of Integrative Zoology, Faculty of Sciences, University of Vienna, 1090 Vienna, Austria; University Museum of Bergen, University of Bergen, Allégaten 41, 5007 Bergen, Norway; School of Biological Sciences, The University of Queensland, Brisbane, QLD 4072 Australia

**Keywords:** Bivalvia, Cephalopod, Complexity, Evolution, Homeobox, Hox, Lophotrochozoa, Neurogenesis, Sensory cell, Polyplacophora

## Abstract

**Background:**

Mollusks represent the largest lophotrochozoan phylum and exhibit highly diverse body plans. Previous studies have demonstrated that transcription factors such as *Pax* genes play important roles during their development. Accordingly, in ecdysozoan and vertebrate model organisms, orthologs of *Pax2/5/8* are among others involved in the formation of the midbrain/hindbrain boundary, the auditory/geosensory organ systems, and the excretory system.

**Methods:**

*Pax2/5/8* expression was investigated by in situ hybridization during the development of representatives of the two major molluscan subclades, Aculifera and Conchifera.

**Results:**

Compared to the investigated polyplacophoran and bivalve species that lack larval statocysts as geosensory organs and elaborate central nervous systems (CNS), cephalopods possess highly centralized brains and statocysts. *Pax2/5/8* is expressed in regions where sensory cells develop subsequently during ontogenesis. Expression domains include esthetes and the ampullary system in polyplacophorans as well as the eyes of cephalopods. No *Pax2/5/8* expression was observed in the less centralized CNS of bivalve, polyplacophoran, and gastropod embryos, thus arguing for a loss of *Pax2/5/8* involvement in CNS development in these lineages. In contrast, *Pax2/5/8* is expressed among others in brain lobes along the trajectory of the esophagus that divides the cephalopod brain.

**Conclusions:**

Our results, along with those on *Otx*- and *Hox*-gene expression, demonstrate that the cephalopod condition is similar to that in mouse and fruit fly, with *Otx* being expressed in the anterior-most brain region (except for the vertical lobe) and a *Pax2/5/8* expression domain separating the *Otx*-domain from a *Hox*-gene expressing posterior brain region. Thus, *Pax2/5/8* appears to have been recruited independently into regionalization of non-homologous complex brains of organisms as different as squid, fruit fly, and mouse. In addition, *Pax2/5/8* is expressed in multimodal sensory systems in mollusks such as the esthetes and the ampullary system of polyplacophorans as well as the eyes of cephalopods. *Pax2/5/8*-expressing cells are present in regions where the future sensory cells such as the polyplacophoran esthetes are situated and hence *Pax2/5/8* expression probably predates sensory cell development during ontogeny. In mollusks, *Pax2/5/8* is only expressed in derivatives of the ectoderm and hence an ancestral role in molluscan ectoderm differentiation is inferred.

## Background

**Fig. 1:**
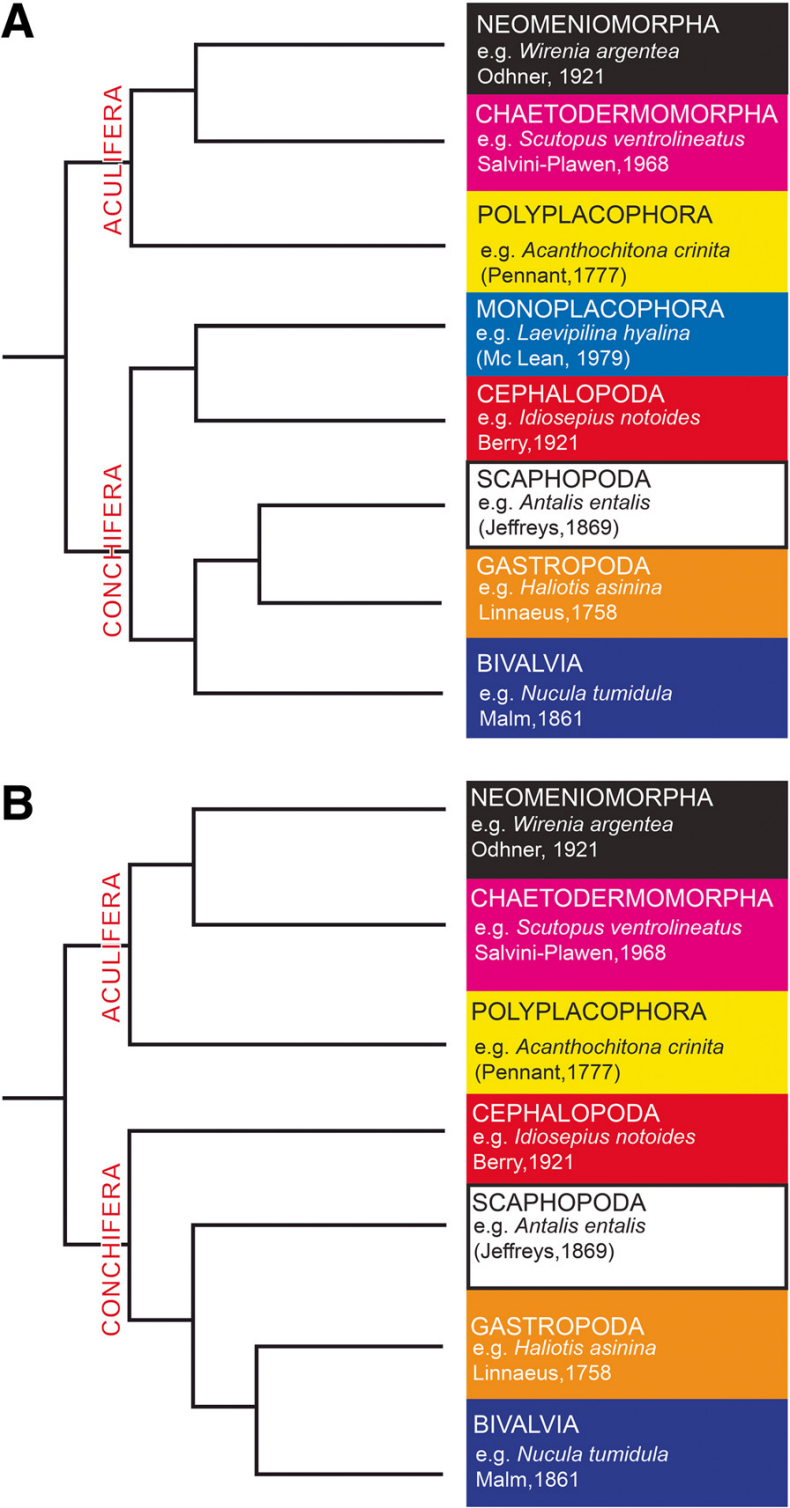
Putative interrelationships of recent mollusks. Two evolutionary scenarios based on recent phylogenomic studies ([**a** 15]; and [**b** 16]); arguing for the Aculifera- Conchifera concept (but see [41] for a different view)

A coordinated tempo-spatial expression of transcription factors is required for cell type specification and the differentiation of the three germ layers into distinct organ systems during bilaterian ontogeny [1]. Studies on ecdysozoan and deuterostome model organisms have elucidated the multifold roles of transcription factors during development; however, a major gap in knowledge does exist for the Lophotrochozoa, one of the three bilaterian superphyla [2]. For inferences concerning the evolutionary conservation versus plasticity of transcription factor expression patterns and their putative roles, a broad comparative approach is imperative. Among the lophotrochozoans, Mollusca is the most speciose phylum and exhibits extremely diverse body plans and adaptative capacities ranging from worm-like spicule-bearing aplacophorans and sessile bivalved clams to highly motile squids (Fig. 1). No less diverse are the ontogenies, with direct and indirect developers and larval forms ranging from trochophore and pericalymma larvae to veligers [3]. For the detection of settlement cues, predator avoidance, location of prey, as well as navigation in a complex environment, the larvae are equipped with sensory organs. The most prominent larval sensory organ is the apical organ that is present in representatives of all molluscan classes investigated so far, except for cephalopods [4–6]. It is equipped with flask-shaped (ampullary) cells and in some mollusks with additional peripheral cells. A so-called ampullary system has been described in polyplacophoran larvae and appears to constitute an apomorphy of the class [5]. It comprises four pairs of sensory cells that are located dorsolaterally and ventrolaterally in the episphere of the trochophore. Cells are of the ampullary sensory cell type, exhibit serotonin-like and FMRFamide-like immunoreactivity and are innervated by the cerebral commissure [5]. Another larval sensory organs, the so-called post-anal organ, only appears to exist in protobranch bivalves [4]. It is composed of sensory cells with two types of cilia and a cavity in proximity to the anus. Coleoid cephalopods do not possess larvae as direct developers but a plethora of sensory organs such as complex camera eyes. These have been subject of a recent study suggesting that a vast number of genes expressed in the squid camera-type eye is not found in the transcriptomes of the nautiloid pinhole eye [7]. Sensory organs as well as other epidermal epithelia or the nervous system are ectodermal derivatives [8]. In mollusks, sensory organs have been described by histological and immunochemical techniques, however, the molecular underpinnings of their development and evolution remain largely obscure (but see [9]).

Members of the paired box (*Pax*) gene family encode transcription factors that are involved in a variety of processes such as the development of excretory organs, the differentiation of the musculature, neuroectoderm specification, and the development of auditory/geosensory systems [10, 11]. In bilaterians, nine orthologous groups of Pax proteins are known that can be distinguished on the basis of the presence or absence of highly conserved structural domains [12]. Members of the Pax2/5/8 group exhibit a conserved N-terminal paired domain, an octapeptide, a partial homeodomain, and a C-terminal transactivation domain [10]. The N-terminal domain and the homeodomain serve as DNA binding sites, the octapeptide is the site for activation or repression of the gene, and the C-terminal domain is the protein/protein interaction domain [10]. In vertebrates and ecdysozoans, *Pax2/5/8* is involved among others in the genesis of the auditory/geosensory system as well as in the formation of the midbrain/hindbrain boundary [13, 14]. For lophotrochozoans, so far only one study has been published on the expression domains and the putative function of *Pax2/5/8* during development. In this study, *Pax2/5/8* expression was found in the statocysts, i.e. the geosensory organ, of the gastropod veliger larva [9].

In order to elucidate putative roles of *Pax2/5/8* during molluscan development, we investigated the expression of its orthologs in representatives of both major molluscan lineages, the Aculifera and the Conchifera [15, 16] (Fig. 1). As a polyplacophoran, *Acanthochitona crinita* has been shown to exhibit putative ancestral features of the Aculifera [17], while the conchiferan *Nucula tumidula* is a protobranch that holds a basal position among the bivalves and possesses a unique pericalymma-type larva [4, 18]. *A. crinita* and *N. tumidula* do not possess statocysts in their larvae and exhibit a less centralized CNS. In contrast, the conchiferan cephalopod *Idiosepius notoides* has a highly centralized CNS and statocysts. This study will test whether *Pax2/5/8* is expressed in ectodermal, endodermal, or mesodermal derivatives during the ontogeny of all three molluscan species and it will shed light on the shared *Pax2/5/8* expression among bilaterian representatives.

## Results

### *Pax2/5/8* gene orthologs and phylogenetic analysis

**Fig. 2:**
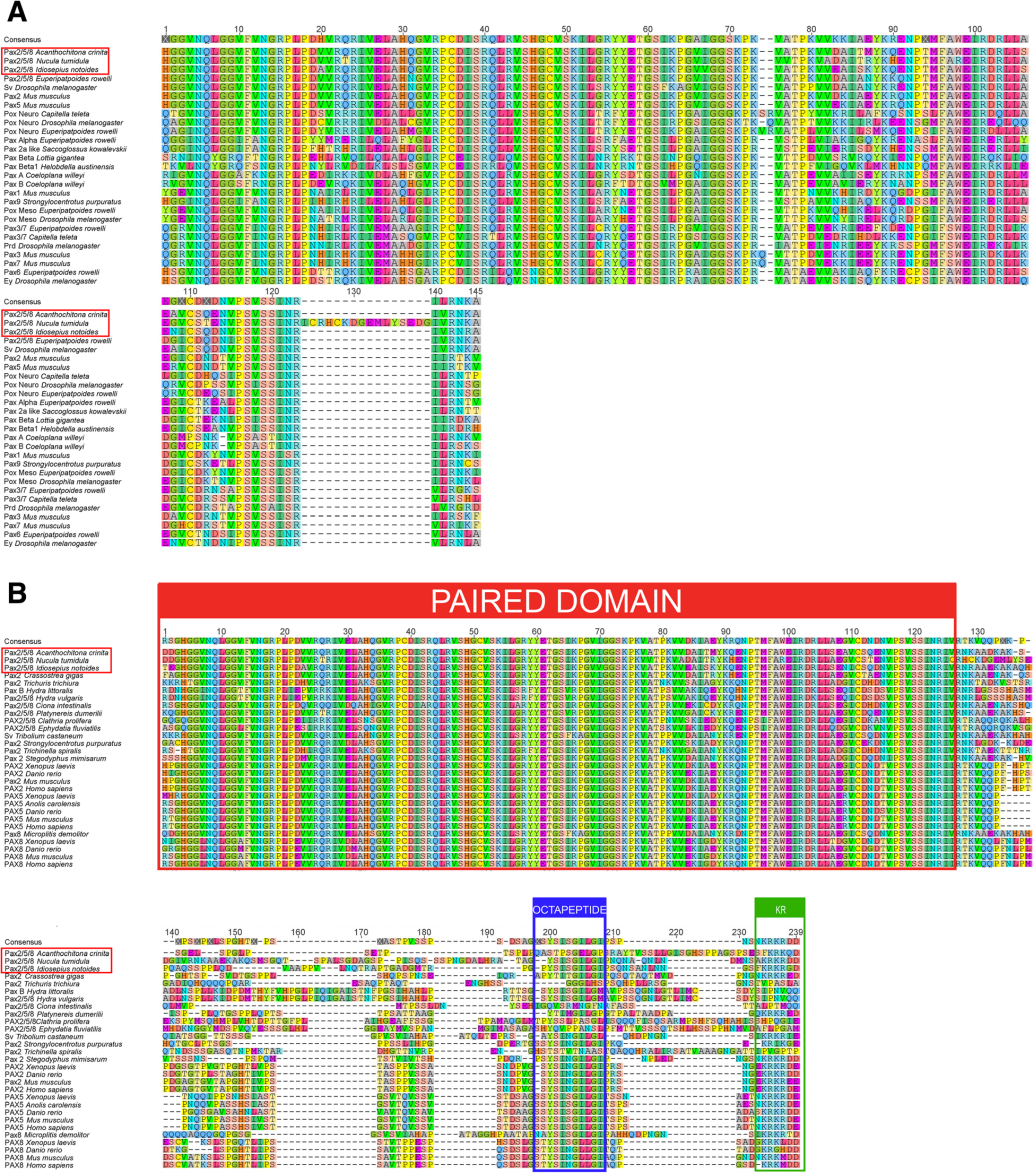
Alignments of the molluscan Pax2/5/8 domains and their bilaterian orthologs. **a** Paired domain alignment of the predicted amino acids of various metazoan Pax genes including Acr-Pax2/5/8, Ntu-Pax2/5/8, and Ino-Pax2/5/8 (highlighted in red). This alignment was used for the maximum-likelihood consensus tree shown in Fig. 3. **b** Alignment of Pax proteins of various metazoans including Acr-Pax2/5/8, Ntu-Pax2/5/8, and Ino-Pax2/5/8 (highlighted in red). The conserved N-terminal paired domain (red), the octapeptide (blue), and the lysine-arginine-rich region (green) are shown, while the partial homeodomain and the C-terminal transactivation domain are omitted. GenBank accession numbers of all encoding genes used are listed in Table 1

**Fig. 3:**
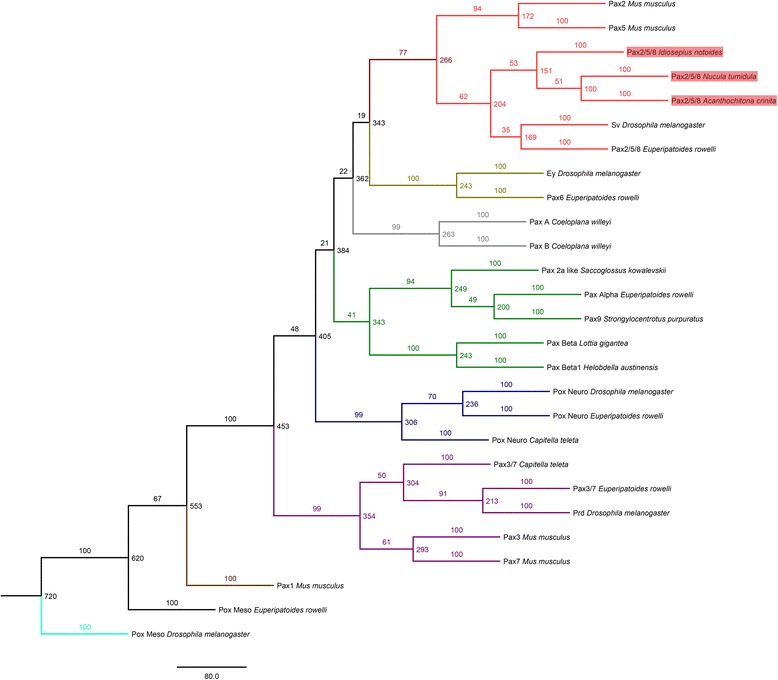
Phylogenetic analysis of Pax proteins. Maximum-likelihood consensus tree with 100 bootstrap replicates constructed from the alignment shown in Fig. 2a. Note that Acr-Pax2/5/8, Ntu-Pax2/5/8, and Ino-Pax2/5/8 (all highlighted in red) cluster with the predicted proteins of their bilaterian *Pax2/5/8* orthologs (red branches/tips). Pax protein families are labeled in different colors

The multiple sequence alignment of Pax2/5/8 orthologs shows the highly conserved N-terminal paired domain, the octapeptide domain, and the lysine-arginine rich region (Fig. 2b). In particular the octapeptide is less conserved among the bilaterian Pax2/5/8 orthologs. The C-terminal partial homeodomain was not included in the alignment since it is even less well conserved. The phylogenetic analysis included proteins of all Pax families and demonstrates that all three molluscan Pax2/5/8 amino acid sequences cluster with their bilaterian orthologs (Fig. 3).

### *Pax2/5/8* expression in the polyplacophoran *Acanthochitona crinita*

**Fig. 4:**
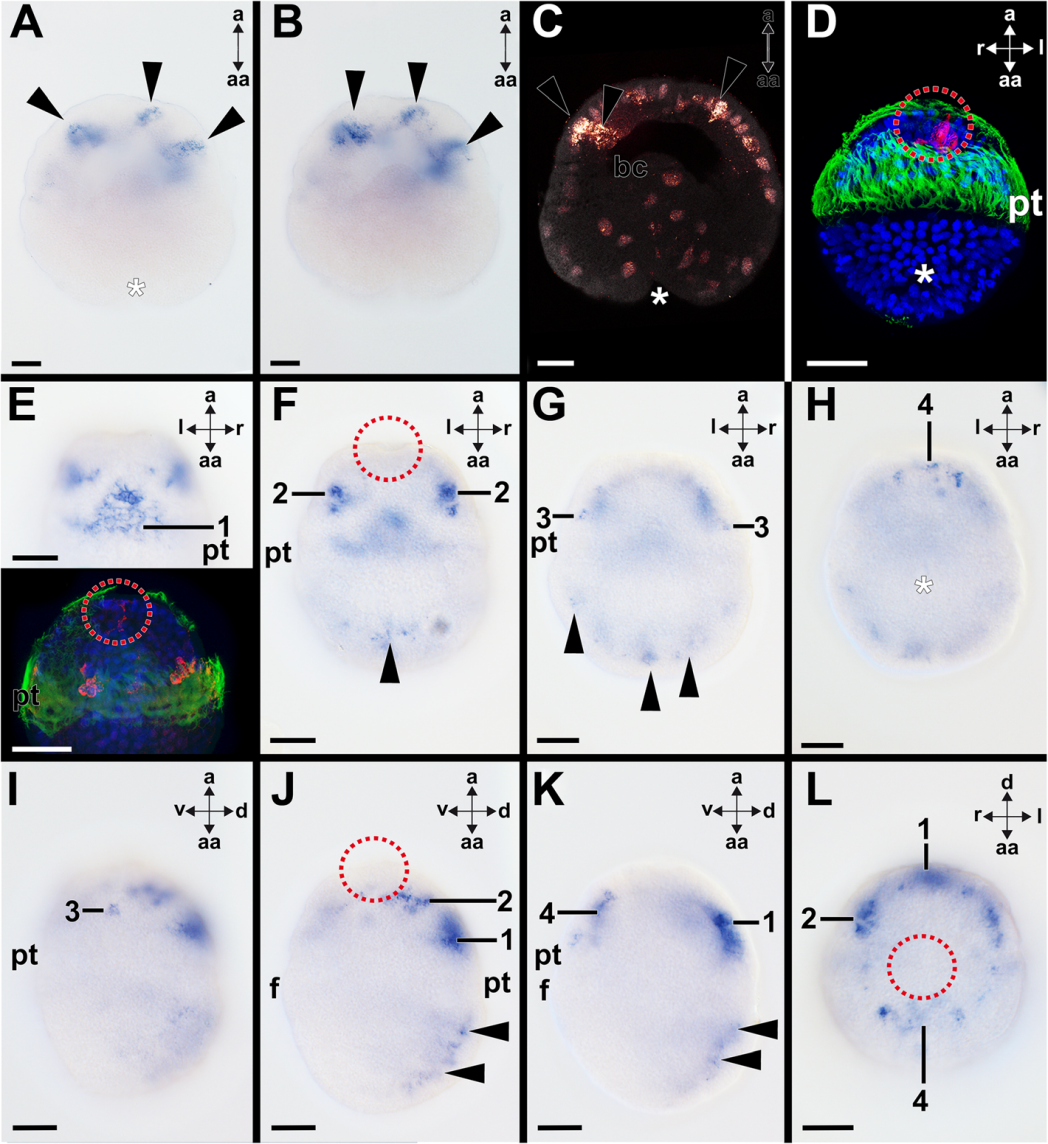
Expression of *Acr-Pax2/5/8* in early larvae of the polyplacophoran *Acanthochitona crinita.* Dorsal (d)–ventral (v), apical (a)-abapical (aa), and left (l)-right (r) axes indicate the orientation. Blastopores/mouth labeled by asterisks and the location of the apical organ is encircled. **a**-**b** Consecutive optical sections through a gastrula showing *Acr-Pax2/5/8*-expressing cells (arrowheads; 3 h post fertilization (hpf)). **c** Confocal reflection scan highlighting same *Acr-Pax2/5/8*-expressing cells (arrowheads) in apical ectoderm as shown in **a**-**b** **d** Three serotonin-like immunoreactive neurons of the apical organ in a 12 hpf early trochophore. **e**-**h** Optical sections from dorsal to ventral through an early trochophore (12 hpf) showing *Acr-Pax2/5/8*-expressing domains 1–4 (termed “group 1-4” in the following) in the episphere and single *Acr-Pax2/5/8*-expressing cells in the hyposphere (arrowheads in (**f**-**g**)). **e** Upper inset: Several *Acr-Pax2/5/8*-expressing cells (1) are located in the ectoderm of the dorsal episphere adjacent to the prototroch (pt). Lower inset: This very early trochophore (10 hpf) exhibits faint FMRFamide-like immunoreactive signal (red staining) in the region of the apical organ and in two groups of four FMRFamide-like immunoreactive ectodermal cells each in the episphere close to the prototroch. **f** Two other bilateral groups of *Acr-Pax2/5/8*-expressing cells (2) are located anterior to group 1 and additional *Acr-Pax2/5/8*-expressing cells lie in the region of the nascent shell fields in the hyposphere (arrowhead). **g** Two *Acr-Pax2/5/8*-expressing ectodermal cells (3) are located bilaterally in the episphere adjacent to the trochoblasts. **h** A group of 5–10 ectodermal *Acr-Pax2/5/8*-expressing cells (4) lies in the central ventral episphere. **i**-**k** Same specimen as shown in **e**-**h** from the left side (**i**) to the mid-sagittal plane (**k**). **l** Apical view of same specimen as shown in **e**-**k** with *Acr-Pax2/5/8* expression domains 1, 2, and 4. Abbreviations: bc, blastocoel; f, foot; pt, prototroch. Scale bars: 20 μm

**Fig. 5:**
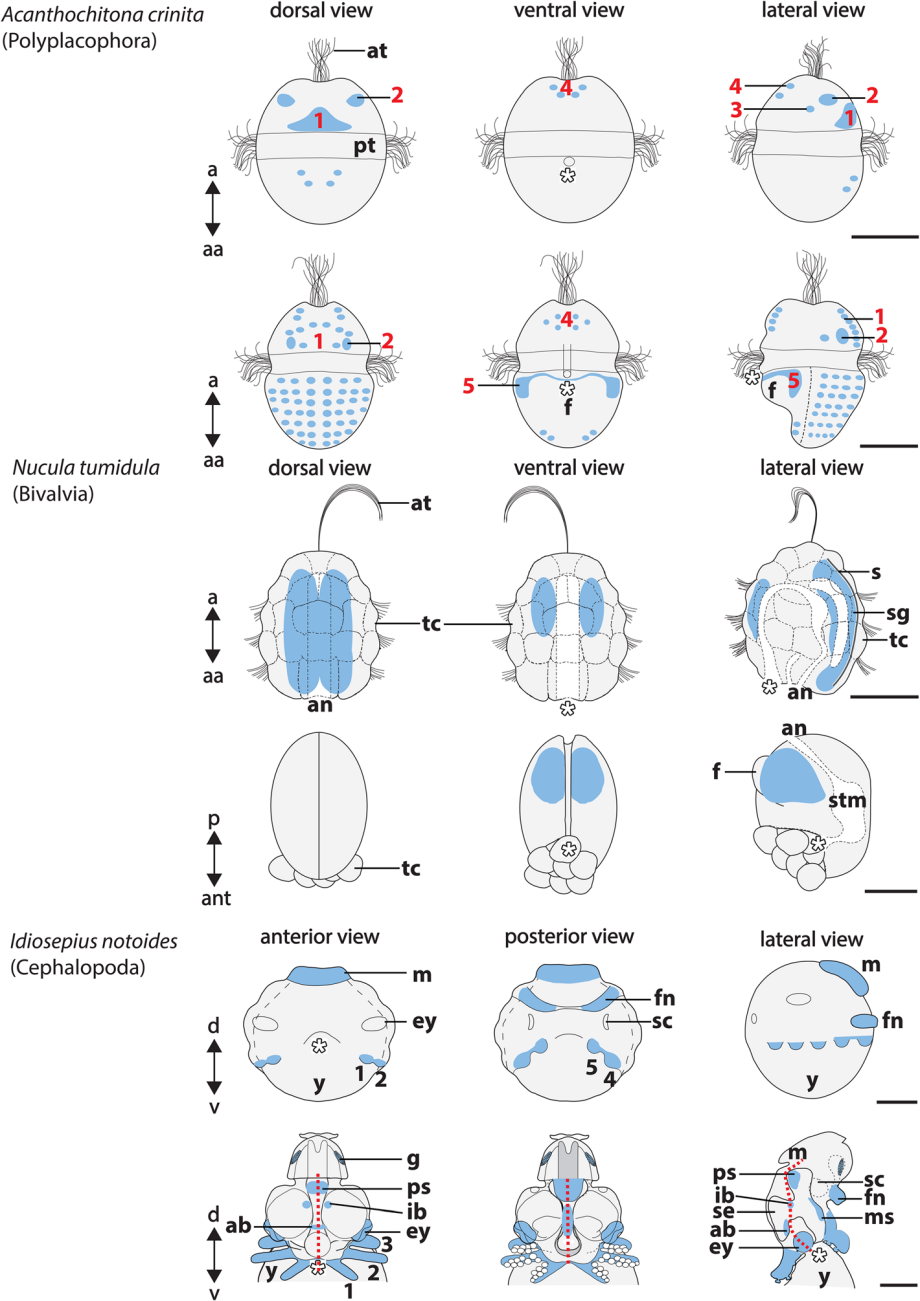
Sketch drawings of *Pax2/5/8* expression in *Acanthochitona crinita*, *Nucula tumidula*, and *Idiosepius notoides.* Dorsal (d)–ventral (v), apical (a)-abapical (aa), and anterior (ant)-posterior (p) axes indicate the orientation. Mouth is marked by asterisk in all panels. *Pax2/5/8*-expressing cell groups are indicated by red numbers in early trochophore larvae of *Acanthochitona crinita* (first row). Additional *Pax2/5/8*-expressing cells are located in the hyposphere of further developed larvae (second row). A pericalymma larva (third row) and a settled individual (fourth row) of *Nucula tumidula* express *Pax2/5/8* in the mantle. Note that *Pax2/5/8* is not expressed in the test cells (tc) that constitute the outermost cell layer. A stage 19 individual (fifth row) and a stage 24–25 individual (sixth row) of *Idiosepius notoides* express *Pax2/5/8* in various domains. Red dashed line indicates trajectory of esophagus and internal yolk. Abbreviations: an, anus; at, apical tuft, ey, eye; f, foot; fn, funnel; g, gill; ib, interbasal lobe; m, mantle; ms, middle subesophageal mass; s, shell; sc, statocyst; sg, shell gland; se, supraesophageal mass; stm, stomach; ps, posterior subesophageal mass; pt, prototroch; y, yolk. Scale bars: 50 μm (except both last scale bars for *I. notoides* with 150 μm)

**Fig. 6:**
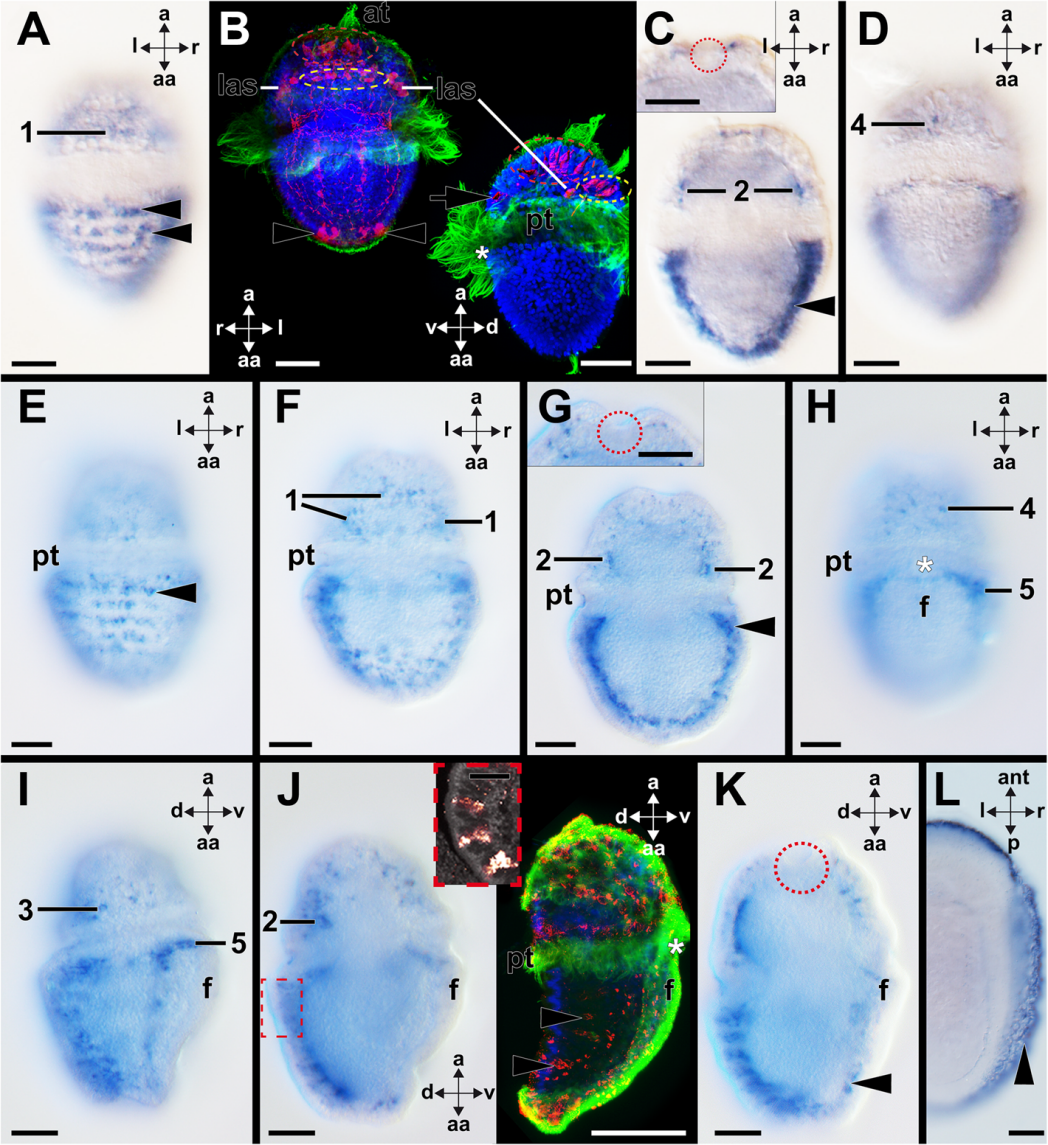
Expression of *Acr-Pax2/5/8* during late larval development and in settled individuals of *Acanthochitona crinita.* Dorsal (d)–ventral (v), apical (a)-abapical (aa), anterior (ant)-posterior (p), and left (l)-right (r) axes indicate the orientation, the red dashed circles the apical organ, and the asterisks label the mouth. Cell nuclei are labeled in blue, cilia in green in B & right inset of J). **a**-**d** *Acr-Pax2/5/8* is expressed in ectodermal cells of the episphere (1), the shell fields (arrowheads in (**a & c**)), the dorso-lateral (2) and ventral episphere (4), and close to the apical organ (inset in **c**) in 35 hpf old trochophores (optical sections from dorsal (**a**) to ventral (**d**)). **b** A further developed, 35 hpf old, trochophore (left micrograph) and a slightly younger specimen (right micrograph) with serotonin-like immunoreactive latero-ventral ampullary cells (las), additional sensory cells (yellow dashed circles), apical organ cells (red dashed circles), and cells in the ventral hyposphere (arrowheads). **e**-**h** In competent trochophores (65 hpf) (optical sections from dorsal (**e**) to ventral (**h**)), *Acr-Pax2/5/8* is expressed in cells of the shell fields (arrowheads), in cells of group 1, 2, and 4 (c.f. Fig. 4), and in two groups of cells (5) along the developing foot (f), which are connected via a slender bridge of cells. Inset in **g** *Acr-Pax2/5/8*-expressing cells close to the apical organ. **i**-**k** Lateral view of trochophore (65 hpf) shown in (**e**-**h**). **j** Dashed box marks location of the small inset that depicts a confocal reflection scan of *Acr-Pax2/5/8*-expressing subepidermal flask-shaped cells (red) in the shell fields. Right large inset: FMRFamide-like immunoreactive cell somata (red) in the shell fields (arrowheads) and close to the trochoblasts. **k** *Acr-Pax2/5/8* expression in the posterior mantle fold (arrowhead). **l** No *Acr-Pax2/5/8* expression was observed in postmetamorphic (settled) specimens (130 hpf). The staining of the perinotum is unspecific (arrowhead). Abbreviations: at, apical tuft; pt, prototroch. Scale bars: 20 μm (except inset in (**j**) 10 μm)

The gastrula stage is reached between 3–5 h post fertilization (hpf) at 20 °C. During this stage *Acr-Pax2/5/8* is expressed in ectodermal cells of the apical region (Fig. 4a–c). Early trochophore larvae hatch at approximately 12 hpf. They are lecithotrophic and are slightly ovoid in shape, with an apical organ and apical tuft in the episphere (Fig. 4d–h). The apical organ comprises three serotonin-like immunoreactive flask-shaped cells and faint FMRFamide-like immunoreactivity (Fig. 4d, e). Two other groups comprising four FMRFamide-like immunoreactive cells each are located in the episphere close to the prototroch (Fig. 4e). The episphere is separated from the hyposphere by two rows of trochoblasts that give rise to the prototroch. *Acr-Pax2/5/8* is expressed in different domains, here referred to as “groups”, located mainly in the larval episphere (Fig. 4e–l). Several *Acr-Pax2/5/8-*expressing cells are located in the ectoderm of the dorsal episphere adjacent to both rows of trochoblasts (group 1 in Figs. 4e, j, k, l and 5). In addition, two other groups are located bilaterally and anterior to group 1 (group 2 in Figs. 4f, j, l and 5). Each group 2 comprises approximately four ectodermal cells that are located subepidermally and project dendrites through the epidermis (group 2 in Figs. 4f, j and 5). Two single *Acr-Pax2/5/8-*expressing cells are located bilaterally, immediately adjacent to the two rows of trochoblasts and more ventrally to group 2 in the episphere (group 3 in Figs. 4g, i and 5). In the center of the ventral episphere, a group of 5–10 ectodermal *Acr-Pax2/5/8*-expressing cells is present (group 4 in Figs. 4h, k, l and 5). The only *Acr-Pax2/5/8* expression domain that is located in the hyposphere of the early trochophore is situated in the region of the nascent shell fields in the dorsal hyposphere (arrowheads in Figs. 4f, g, j, k and 5). The apical organ does not express *Acr-Pax2/5/8* (red dashed circle in Fig. 4f, j, l). In further developed trochophore larvae (35 hpf), *Acr-Pax2/5/8*-expression is less pronounced in the episphere, while *Acr-Pax2/5/8* expression in the hyposphere gains in intensity (Fig. 5; arrowheads in Fig. 6a, c). This and other *Acr-Pax2/5/8*-expressing cell groups are located in similar expression domains as described for earlier developmental stages (Fig. 5; cf. arrowheads in Fig. 4j, k with arrowheads in Fig. 6a, c). *Acr-Pax2/5/8*-expressing cells are present in the expression domains of groups 1, 2, and 4 of previous stages (c.f. Figs. 5 and 6a, c, d). Furthermore, serotonin-like immunoreactive cell somata are located in corresponding regions, such as the lateroventral ampullary cells corresponding to *Acr-Pax2/5/8*-expressing group 2, or the additional sensory cells that are located in the dorsal episphere that correspond to group 1 (Fig. 6b). In the expression domains of *Acr-Pax2/5/8*-expressing groups 3 and 4, FMRFamide-like immunoreactive cell somata are located (not shown). In further developed trochophore larvae (35 hpf), the apical organ does not exhibit *Acr-Pax2/5/8* expression, however, very close to it, *Acr-Pax2/5/8-*expressing cells are located (Fig. 6c). In metamorphic competent trochophore larvae (65 hpf), the *Acr-Pax2/5/8* expression pattern is similar to the one of earlier trochophores (35 hpf) (Figs. 5 and 6e–k). Strong *Acr-Pax2/5/8* expression is present in cells of the nascent shell fields (Figs. 5 and 6e). These *Acr-Pax2/5/8*-expressing cells are located in the epidermis and project dendrites to the periphery (inset in Fig. 6j). In addition, *Acr-Pax2/5/8-*expressing cells are numerous in groups 1–4 during earlier development (Figs. 5 and 6f–j). Interestingly, all these *Acr-Pax2/5/8* expression domains house FMRFamide-like-immunoreactive (usually flask-shaped) cells (Fig. 6j).

An additional *Acr-Pax2/5/8* expression domain is present close to the lateral borders of the foot (Fig. 5, group 5 in Fig. 6h, i). Both groups 5 are interconnected via a thin band of *Acr-Pax2/5/8*-expressing cells that runs along the abapical side of the mouth (Figs. 5 and 6h, i). Few *Acr-Pax2/5/8*-expressing cell are present in the posterior mantle fold (Figs. 5 and 6k), while no *Acr-Pax2/5/8*-expressing cells are located in the region of the apical organ (dashed circle in Fig. 6k).

Settled animals (130 hpf) commence to reduce their episphere and the foot becomes more pronounced. Metamorphosis is largely completed with the shedding of the prototroch, although the eighth shell plate is not formed until probably several weeks after settlement. Early settled animals solely possess few faintly stained *Acr-Pax2/5/8*-expressing cells in the region of the shell fields (Fig. 6l) and only non-specific staining was observed in the perinotum that surrounds the plates (Fig. 6l).

### *Pax2/5/8* expression in the bivalve *Nucula tumidula*

**Fig. 7:**
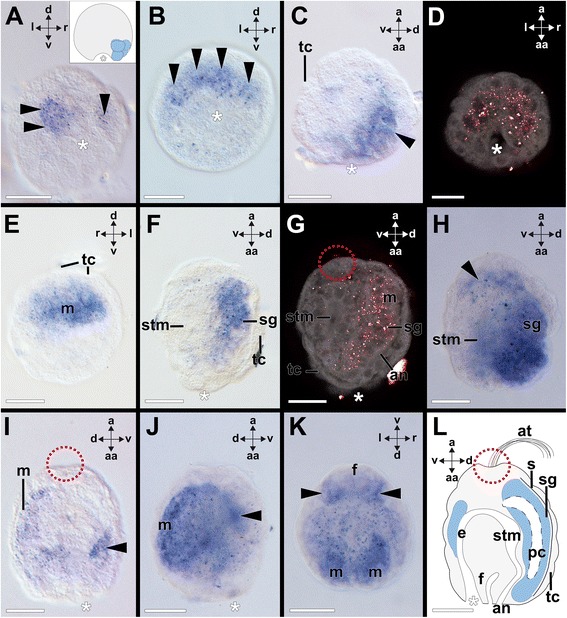
Expression of *Ntu-Pax2/5/8* during larval development of the bivalve *Nucula tumidula.* Dorsal (d)–ventral (v), apical (a)-abapical (aa), and left (l)-right (r) axes indicate the orientation. Blastopore and mouth is marked with an asterisk and the position of the apical organ is encircled. **a** *Ntu-Pax2/5/8* expression in approximately five cells (arrowheads) close to the blastopore of a 24 h post fertilization (hpf) old gastrula. Inset: Sketch drawing of *Ntu-Pax2/5/8* expression (blue) in a gastrula (left view and apical faces up). **b**-**c** 5–7 cells (arrowheads) express *Ntu-Pax2/5/8* below the test cells and close to the mouth of the early pericalymma larva (3 dpf). **d** Confocal reflection (*Ntu-Pax2/5/8* expression in red) and autofluorescence (grey) scan of a single optical section of an early larva (3 dpf; similar to (**b** & **c**)). **e** A further developed specimen (8 dpf) with *Ntu-Pax2/5/8* expression in the mantle (m). **f** *Ntu-Pax2/5/8* is expressed in the mantle including the shell gland (sg) (same specimen as shown in (e). **g** Confocal reflection (*Ntu-Pax2/5/8* expression in red) and autofluorescence (grey) scan of a single optical section of the same specimen as shown in (**f**). **h** This further developed larva (8 dpf) expresses *Ntu-Pax2/5/8* bilaterally along the dorsal and apical mantle (arrowhead). **i** Late larvae (12 dpf) express *Ntu-Pax2/5/8* along the dorsal and ventral mantle (arrowhead). **j** 12 dpf old larva of the same stage as the one shown in I with stronger *Ntu-Pax2/5/8* expression in the dorsal and ventral (arrowhead) mantle. **k** Same specimen as shown in J with *Ntu-Pax2/5/8* expression in the dorsal and ventral mantle (arrowheads) but not the foot (f). **l** Sketch drawing summarizing the *Ntu-Pax2/5/8* expression domains (blue) in a metamorphic competent pericalymma larva before settlement. Abbreviations: an, anus; at, apical tuft; e, esophagus; f, foot; pc, perivisceral cavity; tc, test cell; s, shell; stm, stomach. Scale bars: 30 μm

**Fig. 8:**
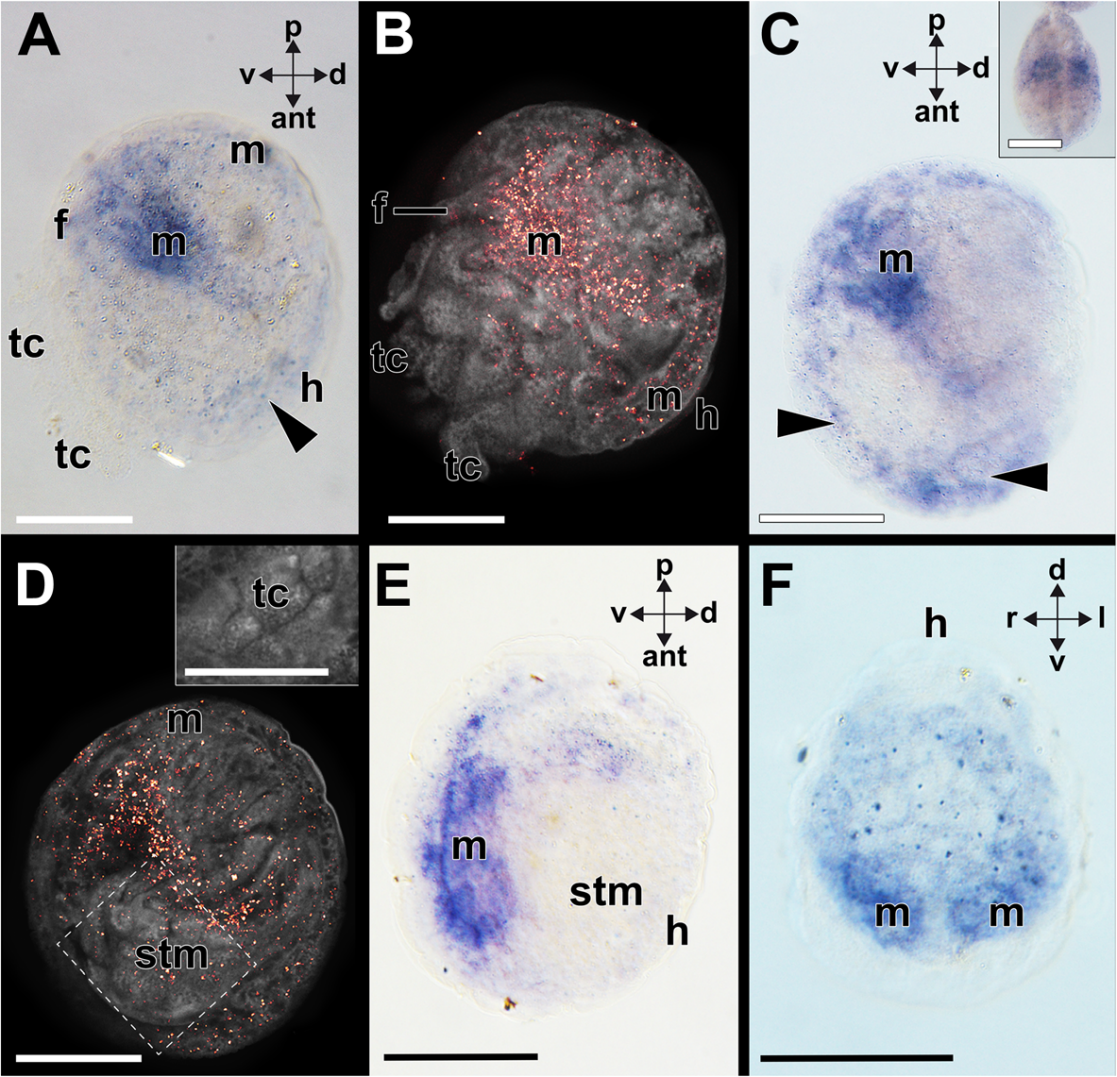
Expression of *Ntu-Pax2/5/8* during postlarval development of the bivalve *Nucula tumidula.* Dorsal (d)–ventral (v), anterior (ant)-posterior (p), and left (l)-right (r) axes indicate the orientation. **a** *Ntu-Pax2/5/8* expression in the mantle of a 22 dpf old larva during onset of metamorphosis. *Ntu-Pax2/5/8* expression is restricted to the mantle (m) margin and a bilateral expression domain located in the postero-ventral central region but not the foot (f). Faint expression (arrowhead) is still visible on the dorsal side of the animal close to the hinge (h). Note the test cells (tc) being ingested by this specimen. **b** Confocal reflection (*Ntu-Pax2/5/8* expression in red) and autofluorescence (grey) scan of a single optical section of the specimen shown in (a) (same orientation). All test cells and the apical organ (not shown) are shed and engulfed during metamorphosis. **c** *Ntu-Pax2/5/8* expression in a settled 22 dpf old individual that has engulfed all test cells. Note the *Ntu-Pax2/5/8* expression along the mantle margin (arrowheads). Inset: Ventral view of a similar staged settled animal. **d** Confocal reflection (*Ntu-Pax2/5/8* expression in red) and autofluorescence (grey) scan of a single optical section of the same 22 dpf old specimen as shown in (c) (same orientation). A different confocal plane of the boxed area is shown in the inset highlighting test cells in the stomach (stm) of the settled larva. **e** *Ntu-Pax2/5/8* expression along the ventral mantle margin in a further developed specimen (22 dpf) with no test cells visible in the stomach (not shown). Note the lack of *Ntu-Pax2/5/8* expression along the dorsal mantle area close to the hinge. **f** Posterior view of the same specimen as shown in E. Scale bars: 50 μm

Embryos of *N. tumidula* reach the 2–4 cell stage by 12 hpf at 6.5 °C and the zygotes gastrulate by approximately 24 hpf. During gastrulation, *Ntu-Pax2/5/8* is expressed in approximately five ectodermal cells close to the blastopore (Fig. 7a). Early pericalymma larvae swim by approximately 48 hpf, are roundish, and possess 5–7 *Ntu-Pax2/5/8*–expressing cells on the future dorsal side below the test-cells (Fig. 7b–d). Slightly further developed pericalymmae are characterized by an apical organ with a ciliary tuft, while mouth and anus are located on the abapical side (Fig. 5). They are covered with large, multiciliary calymma (test) cells. Subsequently, three rows of test cells bear three bands of longer cilia, while shorter cilia still cover the area around the apical tuft as well as the abapical side around mouth and anus. These larvae express *Ntu-Pax2/5/8* in the area of the dorsal mantle (Fig. 7e) and the expression domain also includes the shell gland that develops as an invagination after gastrulation on the future dorsal side (Fig. 7f, g). During subsequent development the apical-abapical axis elongates and the foot develops on the ventral side. Meanwhile, the shell field on the dorsal side has secreted two shell valves. Further developed larvae express *Ntu-Pax2/5/8* in more ventral and apical mantle regions (Fig. 7h). Late pericalymma larvae express *Ntu-Pax2/5/8* along the dorsal mantle region as well as in ventral domains of the mantle (Figs. 5 and 7i–k). Larvae that are metamorphic competent are more ovoid in shape than earlier larvae and express *Ntu-Pax2/5/8* mostly in dorsal domains but also in ventral ones (summarized in Figs. 5 and 7l). Lecithotrophic larvae swim until 15–20 dpf in the water column and finally settle. During the settlement process that may last for several hours, larvae appear to probe the substrate and metamorphosis commences with shedding and ingestion of the test cells and the apical organ (not shown). Larvae express *Ntu-Pax2/5/8* mainly bilaterally in two postero-ventral domains of the central mantle (Figs. 5 and 8a, b). Faint expression is still visible on the dorsal side close to the hinge of the developing valves (Figs. 5 and 8a, b). In individuals that have lost their test cells, *Ntu-Pax2/5/8* is expressed in the posteroventral region of the mantle and along the mantle margin (Figs. 5 and 8c, d). Early juveniles express *Ntu-Pax2/5/8* in lateral portions of the ventral mantle (Figs. 5 and 8e, f).

### *Pax2/5/8* expression in the cephalopod *Idiosepius notoides*

As a typical cephalopod, *I. notoides* is a direct developer with discoidal cleavage. Hatching of the juvenile occurs within 9–10 days after oviposition at 25 °C. In the present study, we adhere to the common designation of embryological orientation in cephalopods, i.e., the mantle apex is considered dorsal and the cephalic region including the brachial crown that surrounds the mouth is considered ventral. The funnel is located posteriorly, while the opposite side including the supraesophageal mass is considered anterior.

**Fig. 9:**
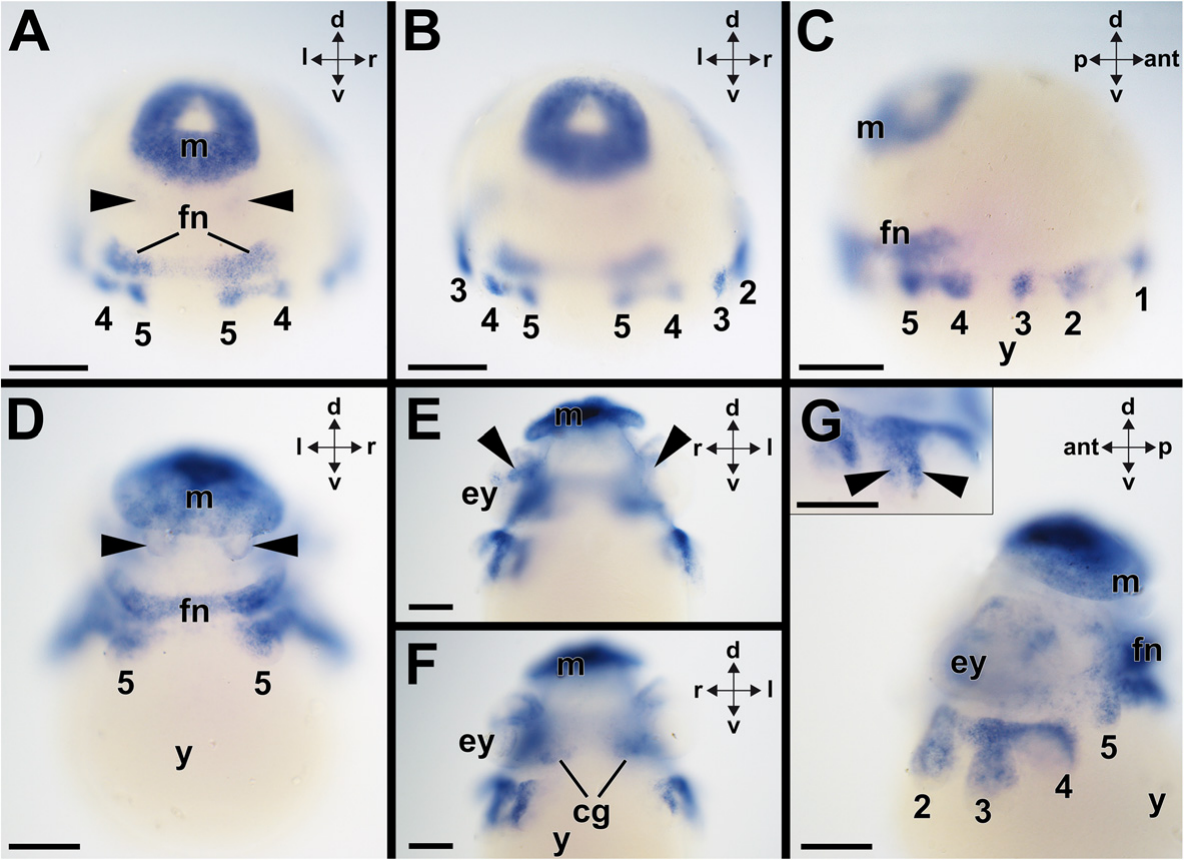
Expression of *Ino-Pax2/5/8* during early prehatching development of the cephalopod *Idiosepius notoides.* Dorsal (d)–ventral (v), anterior (ant)-posterior (p), and left (l)-right (r) axes indicate the orientation. **a** *Ino-Pax2/5/8* is expressed in the mantle (m), the funnel (fn), and all five arm pairs (here only expression in arm pairs 4–5 visible) of stage 19 individuals. Note the faint *Ino-Pax2/5/8* expression in the gills (arrowheads). (**b**) Same specimen as shown in (a) with *Ino-Pax2/5/8* expression in the 2-5^th^ arm pair (2–5). **c** Lateral view of same specimen as shown in (**a**) and (**b**) exhibiting expression of *Ino-Pax2/5/8* in all five arm pairs (1–5). **d** Stage 21 individuals show *Ino-Pax2/5/8* expression in the mantle, the fifth arm pair (5), and the funnel. Note the faint *Ino-Pax2/5/8* expression in the gills (arrowheads). **e** *Ino-Pax2/5/8* expression in portions of the optic ganglia (arrowheads) close to the eyes (ey) in the same specimen as shown in (**d**) **f** Same specimen as shown in (**d**-**e)** with *Ino-Pax2/5/8* expression in the cerebral ganglia (cg). **g** Lateral view of the same specimen as shown in (**d**-**f**) Inset shows detail of *Ino-Pax2/5/8* expression in the arms (arrowheads). Abbreviations: y, yolk sac. Scale bars: (**a**-**g**) 150 μm

**Fig. 10:**
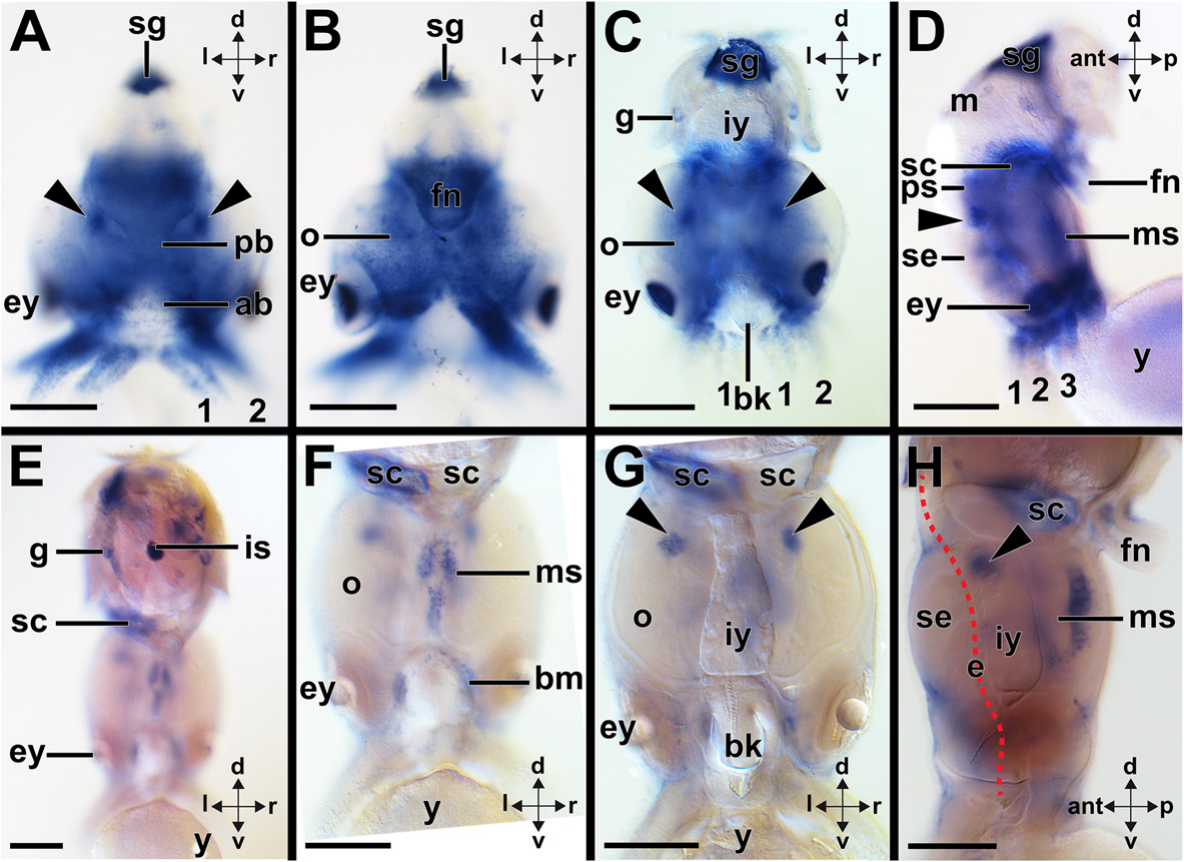
Expression of *Ino-Pax2/5/8* during late prehatching development of the cephalopod *Idiosepius notoides.* Dorsal (d)–ventral (v), anterior (ant)-posterior (p), and left (l)-right (r) axes indicate the orientation. Yolk sac (y) removed in (**a**-**c**) Arms are labeled with numbers. **a** Stage 24 individuals express *Ino-Pax2/5/8* in all arms, the eyes (ey), the epidermis of the cephalopedal region, the interbasal lobes (arrowheads), the posterior basal lobes (pb), and the anterior basal lobes (ab). Note the unspecific staining around the shell gland (sg). **b** Same stage 24 individual as shown in (**a**) Note *Ino-Pax2/5/8* expression in the proximal optic lobes (o), the eyes, and the funnel (fn). **c** *Ino-Pax2/5/8* is expressed in smaller portions of the body of stage 25 individuals compared to earlier stage individuals. *Ino-Pax2/5/8* is expressed in the gills (g), the eyes, and in the proximal but not the distal arm portions. Note *Ino-Pax2/5/8* expression in the interbasal lobes (arrowheads). **d** The interbasal lobes (arrowhead) and anterior basal lobes of the supraesophageal mass (se) express *Ino-Pax2/5/8*. The posterior perikaryal layer of the middle subesophageal mass (ms) as well as the posterior subesophageal mass (ps) express *Ino-Pax2/5/8. Ino-Pax2/5/8* expression could not be observed in the distal funnel of this stage 25 individual. Please note the unspecific staining around the statocyst (sc). **e**-**h** In stage 27 individuals, *Ino-Pax2/5/8* is restricted to few expression domains such as the gills, the posterior perikaryal layer of the middle subesophageal mass, the interbasal lobes (arrowheads), tissue around the buccal mass (bm), and the base of the funnel. Trajectory of esophagus is indicated by red dashed line in (**h**) Abbreviations: bk, beak; is, ink sac; iy, internal yolk; se, supraesophageal mass. Scale bars: (**a**-**d**) 150 μm; E-H: 200 μm

Anlagen of the CNS, the arms, the funnel, and the mantle are discernable from stage 18 onward (for a common staging system, see [19]). Early stage 19 individuals of the pygmy squid *I. notoides* express *Ino-Pax2/5/8* in the mantle, the funnel, and all ten arms (Figs. 5 and 9a–c). Faint *Ino-Pax2/5/8*-expression is present in the developing gills of stage 19 individuals (Fig. 5; arrowheads in Fig. 9a). During subsequent development, *Ino-Pax2/5/8* expression is located in the same domains as in stage 19 individuals (Fig. 9d–g). *Ino-Pax2/5/8* is additionally expressed in the tissue that interconnects the arms (Fig. 9g) and in nervous tissue of the optic ganglia (arrowheads in Fig. 9e). Stage 24 individuals strongly express *Ino-Pax2/5/8* in all arms, the funnel, eyes, and the epidermis of the cephalopedal region (Figs. 5 and 10a, b). Additionally, *Ino-Pax2/5/8*-expressing cells are present in the region of the interbasal lobes that both belong to the posterior basal lobes of the brain (Fig. 5; arrowheads in Fig. 10a). During stages 24–25, *Ino-Pax2/5/8* is also expressed in the eyes (Figs. 5 and 10b, c). Stage 25 individuals still express *Ino-Pax2/5/8* in the gills and in large areas of the epidermis of the cephalopedal region (Fig. 10c, d). *Ino-Pax2/5/8* expression is only located in the proximal portions of all arms and the proximal portion of the funnel (Fig. 10c, d). *Ino-Pax2/5/8* expression domains in the CNS include the anterior basal and the interbasal lobes that both belong to the supraesophageal mass (Fig. 10d). The vertical lobe complex as highest integration center does not express *Ino-Pax2/5/8* (Fig. 10d). *Ino-Pax2/5/8* is also faintly expressed in the posterior subesophageal mass (Fig. 10d). The posterior perikaryal layer of the middle subesophageal mass also expresses *Ino-Pax2/5/8* (Fig. 10d). In late prehatching developmental stages such as stage 27, *Ino-Pax2/5/8* is expressed in the gills (Fig. 10e), in the posterior perikaryal layer of the middle subesophageal mass, and around the buccal mass (Fig. 10f). *Ino-Pax2/5/8* expression in the interbasal lobes persists in stage 27 individuals (Fig. 10g, h). Faint *Ino-Pax2/5/8* expression is still present at the base of the funnel (Fig. 10h).

## Discussion

### Evolution of Pax2/5/8 proteins

Our phylogenetic analysis confirms the identities of all three molluscan Pax2/5/8 proteins presented herein. The sequences of *Acanthochitona crinita*, *Idiosepius notoides*, and *Nucula tumidula* exhibit a paired domain, an octapeptide, the lysine-arginine rich region, as well as the partial homeodomain (not shown) diagnostic for *Pax2/5/8*.

### *Pax2/5/8* is expressed in the development of multimodal sensory systems of mollusks

Among bilaterians, *Pax2/5/8* expression is particularly well-investigated in the Deuterostomia and Ecdysozoa [13, 20]. Studies on various deuterostomes revealed that orthologs of *Pax2/5/8* play a role during development of auditory/ geosensory organs [21–23]. Interestingly, the phylogenetically distantly related gastropod mollusk *Haliotis asinina* also expresses *Pax2/5/8* in its geosensory organ, the statocysts [9]. This present study demonstrated that homeobox genes may be recruited into the development of organs with similar function but of different evolutionary origin (i.e., in non-homologous systems). It also poses the question as to how *Pax2/5/8* is recruited during the ontogeny of organisms without auditory and equilibrial sense.

In our study, we included the chiton *Acanthochitona crinita* and the bivalve *Nucula tumidula*, both lophotrochozoan representatives without larval statocysts, in order to assess the role of *Pax2/5/8* during their development. *Pax2/5/8* is expressed in several groups of ectodermal cells in the episphere and the hyposphere of the polyplacophoran trochophore. Although *A. crinita* and *N. tumidula* possess larval apical (sensory) organs, no *Pax2/5/8* expression was detected in components of this organ. In *A. crinita* cells of groups 2–3 are located in a similar location as cells of the larval ampullary sensory system that is an autapomorphy of Polyplacophora (Figs. 5 and 6 in present study; [5]). Cells of the ampullary system are flask-shaped and probably chemosensory rather than mechanosensory [5]. *Pax2/5/8* expression and FMRFamide-like immunoreactivity may be co-localized in ectodermal cells in the dorsal episphere adjacent to the prototroch (group 2, c.f. Figs. 5 and 6j). Further *Pax2/5/8*-expressing ectodermal cells (group 4) are located in the ventral episphere and might correspond to the four serotonin-like immunoreactive flask-shaped cells (c.f. group 4 in Figs. 4h, 5, 6b). The immunochemical and molecular phenotype, their flask-shaped somata, and their subepithelial location argue for a sensory role of the above-mentioned cells [5]. This might also be true for the *Pax2/5/8-*expressing groups 5 that are located on the ventral side along the borders of the foot of advanced trochophore larvae (Fig. 5). Both groups are connected to each other via a small band along the abapical side of the mouth/ apical side of the foot (Fig. 5). This expression domain also includes a region where the “Schwabe organ”, a putative chemosensory organ of a polyplacophoran ingroup, is located [24]. Although the chitonid *A. crinita* has not been studied with respect to the presence of this organ, it is likely that it is not present in *A. crinita* since other congeneric species of the Chitonida lack this sensory structure. *Pax2/5/8* expressing cells are also located in the posterior mantle fold that exhibits the chemosensory osphradia of adult chitons (arrowheads in Fig. 6k; see also [24]. In both described expression domains, however, no *Pax2/5/8* expression is found in postmetamorphic chitons.

A prominent group of *Pax2/5/8*-expressing cells is present in the shell fields of *A. crinita* (arrowheads in Figs. 4j, k, 5 and 6a). The majority of these cells is located in central regions of each shell field and exhibits processes that penetrate the epidermis and the nascent calcareous layers of the shell plates (inset in Fig. 6j). These processes exhibit FMRFamide-like immunoreactivity in earlier developmental stages than the associated cell somata (c.f. Figs. 4j, 5 and 6a, j). Judging by their location and morphology, these *Pax2/5/8*-expressing and FMRFamide-like immunoreactive cells are probably esthetes. Esthetes are primarily photoreceptive organs that constitute a polyplacophoran autoapomorphy and penetrate the surface of the shell plates of adult specimens [25–27]. During ontogeny, esthetes may first be identified by the narrow holes of their canals in shell plates of metamorphosed specimens [28].

In the pygmy squid, *Pax2/5/8* is expressed in various organs that are known to be equipped with sensory cells. During a short developmental time frame (stages 24–25), *Ino-Pax2/5/8* is expressed in the eyes (Figs. 5 and 10b, c), resembling the situation of the adult gastropod *Haliotis asinina* [29]. The brachial crown, i.e. all five arm pairs, expresses *Pax2/5/8* in epidermal cells until stage 24 (Fig. 10a, b). In stage 25 individuals, *Pax2/5/8* expression is restricted to the proximal part of each arm/tentacle. Expression fades in subsequent developmental stages.

Cephalopod arms and tentacles house a plethora of sensory cells. Although cell proliferation experiments during cephalopod arm growth are still lacking, it is tempting to speculate that *Pax2/5/8* is expressed in ectodermal sensory cells in regions of high proliferation activity, such as the proximal portion of the brachial crown in stage 25 specimens. The funnel shows a similar pattern as the brachial crown. During early development the entire funnel expresses *Pax2/5/8*, while only the proximal portion expresses *Pax2/5/8* in subsequent stages until it fades entirely in late prehatching stages. Here, *Pax2/5/8* appears to be expressed in the epidermis as it has also been reported for vertebrates [30]. Along the lateral sides of the foot the future gills develop and *Pax2/5/8* expression predates their development.

### Expression of *Pax2/5/8* in the molluscan mantle

*Pax2/5/8* is expressed in different regions of the developing mantle of the aculiferan polyplacophoran *Acanthochitona crinita*, the basal bivalve *Nucula tumidula*, and the cephalopod *Idiosepius notoides*. This corroborates data on the veliger larva of *Haliotis asinina*, where *Pax2/5/8* is expressed in the anterior region of the mantle that is known to be richly equipped with sensory cells [9]. In contrast to the esthetes as distinct expression domain of *A. crinita, Pax2/5/8* is rather broadly expressed in the mantle of *N. tumidula* and *I. notoides*. During earlier development of *N. tumidula*, *Pax2/5/8* expression is, however, first restricted to the dorsal mantle epithelium, including the shell gland. In contrast, the shell gland of *H. asinina* does not exhibit *Pax2/5/8* expression (present study and [9]). Shortly before metamorphosis, *Pax2/5/8* expression shifts to the ventral side of the mantle and disappears in the dorsal region after metamorphosis. This may be explained by the onset of sensory cell development on the ventral side of the mantle that is exposed to water currents in the postmetamorphic, settled animal. Other ectodermal expression domains comprise the cephalopedal epidermis of stage 24 individuals of *I. notoides* (Fig. 10a).

### *Pax2/5/8* plays a role in cephalopod brain regionalization

**Fig. 11:**
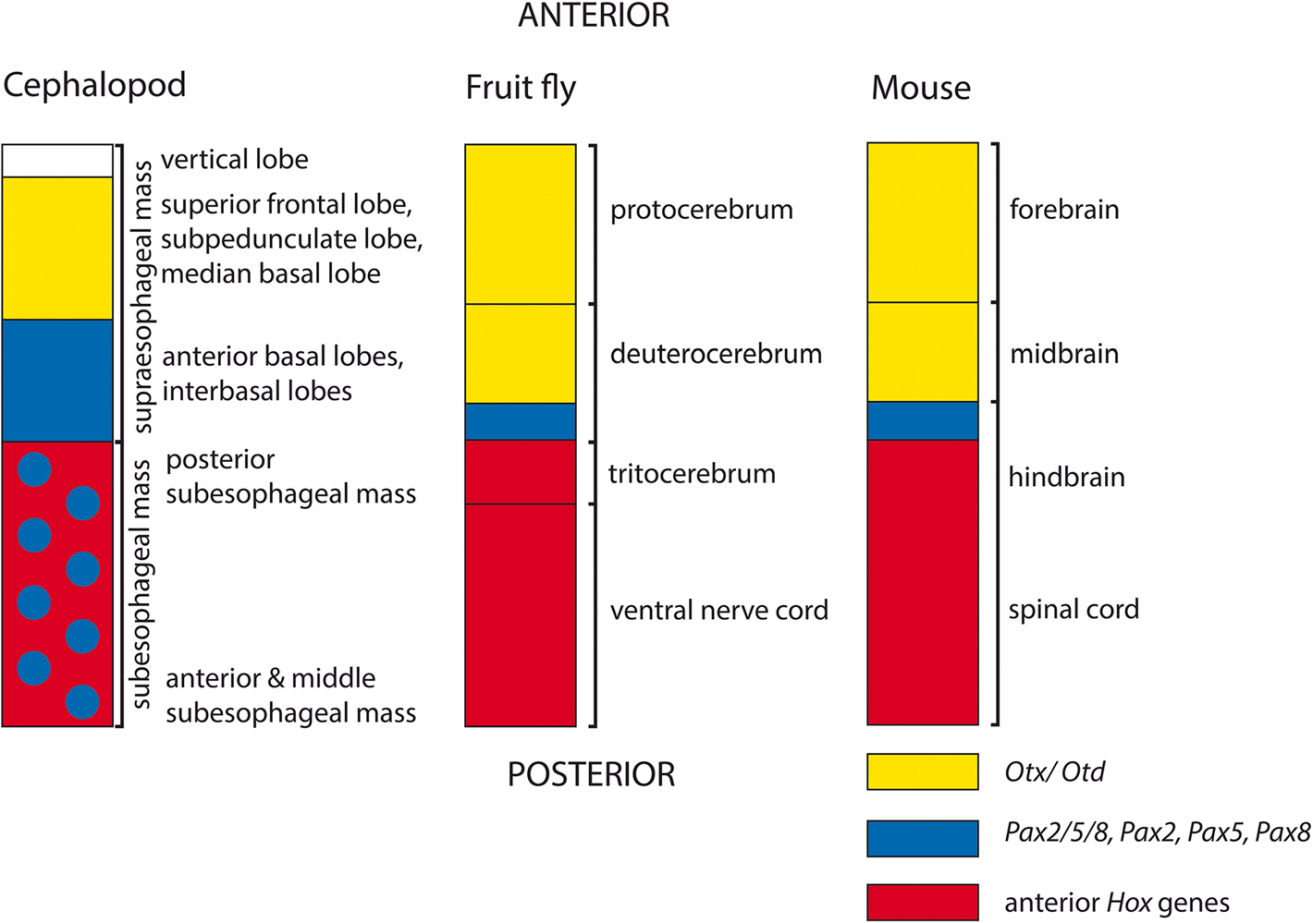
*Pax2/5/8* has been independently recruited in brain regionalization in various bilaterian subgroups. Expression of *Otx*, *Pax2/5/8*, and anterior *Hox* genes during brain formation in a lophotrochozoan (cephalopod), an ecdysozoan (*Drosophila melanogaster*), and a deuterostome (*Mus musculus*). Anterior-most brain regions face up and posterior-most brain regions face down. The cephalopod vertical lobe does not express *Otx*, *Pax2/5/8*, or *Hox* genes. The superior frontal, subpedunculate, and median basal lobes express *Otx* in *Sepia officinalis* [31] and the anterior basal and interbasal lobes express *Pax2/5/8* in *Idiosepius notoides* (present study). The posterior subesophageal mass and the posterior perikaryal layer of the middle subesophageal mass express *Pax2/5/8* in *I. notoides* (present study). Anterior *Hox* genes are expressed in the palliovisceral and pedal ganglia/ subesophageal mass of other cephalopods [32]. Sketch modified after [14]

Studies on mouse and *Drosophila* embryos revealed that several gene orthologs play a role in brain regionalization. The anterior-most brain regions of both organisms, i.e. the murine forebrain/midbrain as well as the fruit fly’s protocerebrum/deuterocerebrum, express *Otd/Otx* (Fig. 11; [14]). The adjacent more posterior brain regions, that are patterned in a spatially collinear fashion by anterior *Hox* gene orthologs, are separated from the anterior-most brain regions by a *Pax2/5/8* expression domain (“midbrain/hindbrain boundary”; Fig. 11; [14]). Interestingly, a similar molecular fingerprint is manifested in the developing brain of the cephalopod *Idiosepius notoides* (present study) and other cephalopods investigated. Here, *Pax2/5/8* expression is restricted to the cerebral ganglia during early development. In subsequent developmental stages transcripts are restricted to the anterior basal and the interbasal lobes that are located slightly anterior to the esophagus, thus dividing the cephalopod brain into a supraesophageal and a subesophageal mass (Fig. 11). Anterior portions of the posterior subesophageal mass that are located close to the esophagus also exhibit faint *Pax2/5/8* expression. Notably, brain lobes that are located more anteriorly, such as the median basal lobe or the vertical lobe complex, do not express *Pax2/5/8* in *I. notoides*. In late-stage prehatching individuals, *Pax2/5/8* is only expressed in the interbasal lobes as well as in the posterior perikaryal layer of the middle subesophageal mass.

*Otx* is expressed in the supraesophageal mass of the cuttlefish *Sepia officinalis* and the pygmy squid *I. notoides* (Fig. 11; [31]; Wollesen, unpublished data). Compared to *Pax2/5/8* it is, however, expressed in more anterior regions of the supraesophageal mass; i.e., in components of the vertical lobe complex (i.e. the superior frontal and subpedunculate lobe) and the median basal lobe of the supraesophageal mass. In contrast to *Pax2/5/8* expression, no *Otx* expression was found in the subesophageal mass (present study, [31], Wollesen, unpublished data). In cephalopods, *Hox* genes are expressed in the CNS with exception of the cerebral ganglia/supraesophageal mass [32]. Although no collinear spatial expression is reported from cephalopods, the *Otx-* and *Pax2/5/8* expression patterns adhere to the overall pattern reported for *Drosophila* and mouse [14]. Data on other bilaterians suggest a *Six3* expression domain in the anterior-most region of the cephalopod brain, i.e. the vertical lobe complex (white box in Fig. 11; [33]). Surprisingly, *Pax2/5/8* is not expressed in the developing nervous system of the polyplacophorans, bivalves, or gastropods investigated so far (present study; [9]). In contrast to cephalopods, the fruit fly, or mouse none of the latter mollusks possess a highly centralized brain.

### *Pax2/5/8* expression is restricted to the ectoderm in mollusks

**Fig. 12:**
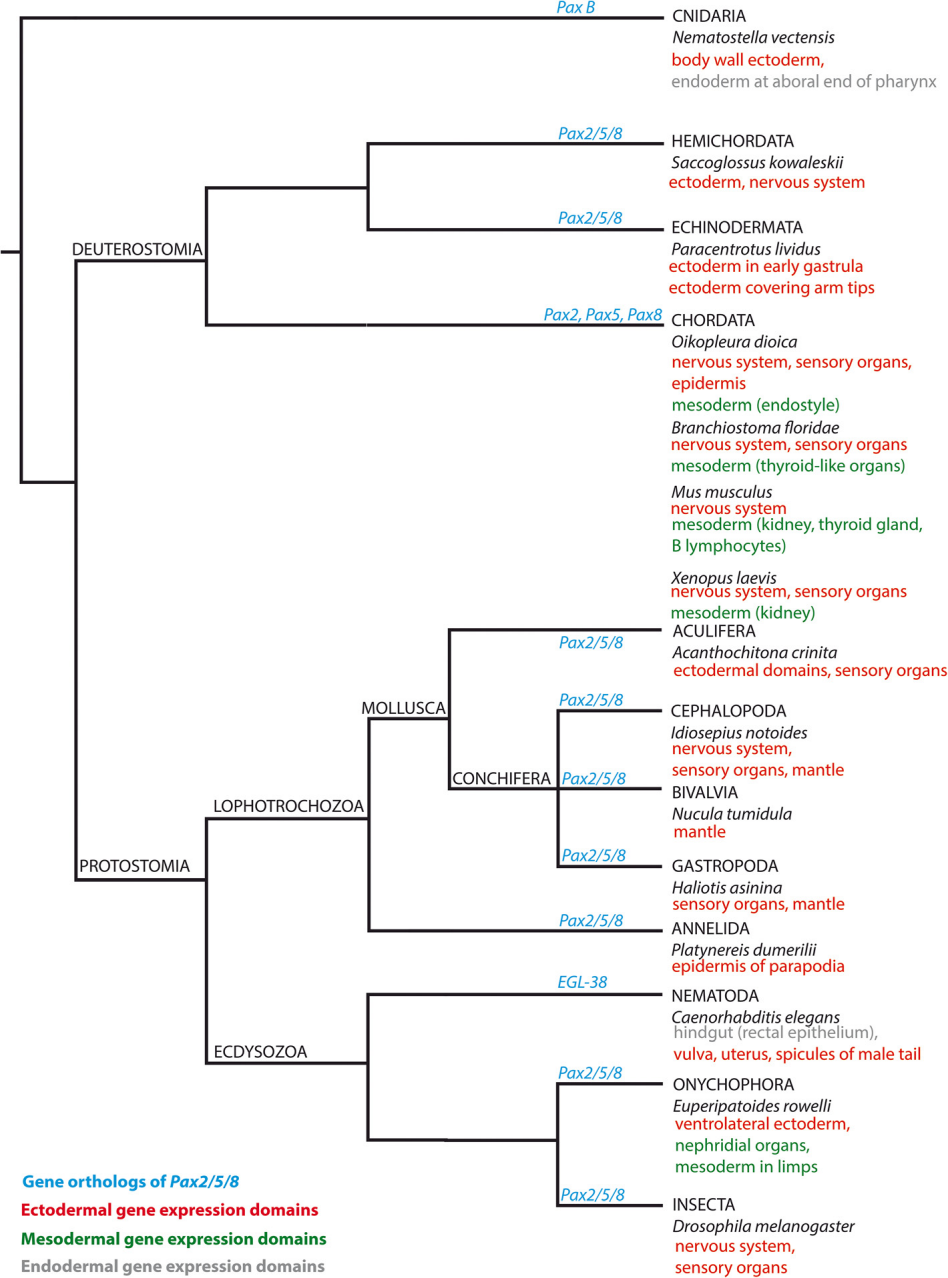
*Pax2/5/8* expression domains during ontogeny of various metazoan representatives. A comparison among metazoan animals suggests that the ancestral role of *Pax2/5/8* was probably restricted to ectodermal domains while chordates and onychophorans recruited orthologs into the development of mesodermal organ systems. The few data on Lophotrochozoa suggest that *Pax2/5/8* expression adheres to the ancestral state, i.e. patterning of certain ectodermal domains. In addition, they show that among mollusks *Pax2/5/8* apparently lost its role in the formation of the nervous system (*Acanthochitona crinita*, *Nucula tumidula*, *Haliotis asinina*) with exception of the cephalopods, while the mantle including shell gland strongly express *Pax2/5/8*. Data on *Pax2/5/8*, *Pax2*, *Pax5*, *Pax8*, *PaxB*-expression: [42] (Cnidaria: *Nematostella vectensis*); [43] (Hemichordata: *Saccoglossus kowalevskii*); [44] (Echinodermata: *Paracentrotus lividus*); [10, 13, 14, 20, 45, 46] (Chordata: *Oikopleura dioica*; *Mus musculus*, *Xenopus laevis*, *Brachiostoma floridae*); present study (Aculiferan mollusks: *Acanthochitona crinita*); [9], present study (conchiferan mollusks: *Haliotis asinina*, *Nucula tumidula*, *Idiosepius notoides*); [34] (study on the regenerating adult animal, Annelida: *Platynereis dumerilii*); [47] (Nematoda: *Caenorhabditis elegans*); [48] (Onychophora: *Euperipatoides rowelli*); [14, 49] (Insecta: *Drosophila melanogaster*). Cladogram simplified after [50]

Among lophotrochozoans, *Pax2/5/8* expression during development has only been studied in mollusks (present study; [9]; but see [34] on the role of *Pax2/5/8* in regenerating tissue of the adult polychaete *Platynereis dumerilii*). In all mollusks investigated so far, *Pax2/5/8* is exclusively expressed in ectodermal domains such as the epidermis, the shell gland, the mantle, sensory cells, as well as the nervous system (present study; [9]). Considering that *Pax2/5/8* is consistently expressed in ectodermal epithelia in all metazoans investigated so far, with solely the Onychophora and the Chordata expressing *Pax2/5/8* in mesodermal domains such as the nephridia, the most parsimonious explanation is an independent recruitment of *Pax2/5/8* in mesodermal epithelia development in these clades (Fig. 12). In nematodes and cnidarians the *Pax2/5/8* orthologs are involved in the formation of endodermal structures such as the hindgut or the endoderm at the aboral end of the pharynx (Fig. 12). Since the anterior-most and posterior-most portions of the digestive tract are located at the endodermal-ectodermal boundary, the *Pax2/5/8* expression domains in the nematode hindgut and the aboral end of the pharynx of the cnidarian *Nematostella vectensis* might be an evolutionary reminiscent of ancestral ectodermal expression domains in the ancestors of these taxa (see Fig. 12).

## Conclusions

The present study shows that *Pax2/5/8* is expressed in multimodal sensory systems in mollusks such as the esthetes and the ampullary system of polyplacophorans, the eyes of squids, but not in the larval apical organ of *A. crinita* and *N. tumidula. Pax2/5/8* expression probably predates sensory cell development during ontogenesis, since *Pax2/5/8*-expressing cells are present in regions where the future sensory cells are situated. Compared to other bilaterians, *Pax2/5/8* is not expressed in the development of the less centralized nervous system of bivalves, gastropods, and polyplacophorans, and hence it most likely lost its role in brain development in these mollusks. Interestingly, *Pax2/5/8* is expressed along the trajectory of the esophagus that divides the cephalopod brain into a supraesophageal and a subesophageal mass. Together with *Otx* and *Hox* genes *Pax2/5/8* might have been recruited into brain regionalization, thus representing an extreme case of convergent evolution of gene function to the situation found in vertebrates (mouse) and insects (fruit fly). Since *Pax2/5/8* is largely expressed in ectodermal domains throughout the Bilateria, its ancestral role was most likely in the differentiation of this outer most germ layer of bilaterians.

## Methods

### Collection and culture of animals

Adults of the polyplacophoran *Acanthochitona crinita* were collected in the intertidal zone close to the Station Biologique Roscoff in Roscoff, France, during the summers of 2013 and 2014. Adults were kept at 20 °C in running seawater. Prior to spawning, adults were isolated and maintained separately in glass dishes. Spawning was induced by exposing adults to sunlight for 3–4 h or thrice each for 15 min to alternating water temperatures, i.e. 25–30 °C and 10–15 °C. Released sperm and oocytes were separated and the latter rinsed several times in Millipore-filtered seawater (MFSW) and fertilized immediately. Early cleavage stages were kept at 20 °C in running seawater, while post-gastrulation stages were kept in MFSW with the addition of antibiotics against bacterial and fungal growth (50 mg streptomycin sulfate (Sigma-Aldrich) and 60 mg penicillin (Sigma-Aldrich) per liter MFSW). Water was changed every other day and settlement of metamorphic competent larvae was induced by the addition of substrate on which adult animals had been found.

Adults of the protobranch bivalve *Nucula tumidula* were collected from sediment that was sampled with a hyperbenthic sled at 180–220 m depth on muddy seafloor in Hauglandsosen (Bergen, Norway) during the winters 2012 and 2013. Adult individuals were kept at 6.5 °C in MFSW (UV-treated) at the marine living animal facilities of the Department of Biology, University of Bergen. *N. tumidula* is dioecious and spawning of gametes was induced by keeping adults in seawater of alternating temperatures. Accordingly, adults were exposed thrice to water of 10–15 and 2 °C for 10 min each. Males that released sperm were separated immediately to avoid polyspermy and the oocytes were rinsed several times in MFSW. Oocytes were fertilized and developmental stages were cultured at 6.5 °C in MFSW in glass bowls with water changes every other day.

Adults of the pygmy squid *Idiosepius notoides* were dip-netted in the sea grass beds of Moreton Bay, Queensland, Australia. Adults were kept in closed aquaria facilities at the School of Biological Sciences of the University of Queensland in Brisbane and fed with various crustaceans. Fertilization is internal and females attached egg clutches to sea grass or to the glass surface of the aquaria. Embryos were cultured and staged as described previously [35].

### RNA extraction and fixation of animals

Several hundred individuals of early cleavage stages, larvae, metamorphic competent individuals to early juveniles of *Acanthochitona crinita* and *Nucula tumidula* were collected and stored in RNAlater (Lifetechnologies, Vienna, Austria) at −20 to −80 °C. RNA was extracted with a RNA extraction kit (Qiagen, Roermond, Netherlands) and stored at −80 °C. For *I. notoides*, egg jelly and chorion of embryos were removed and RNA from approximately 300 specimens including freshly laid zygotes (stage 1) to hatchlings (stage 30) was extracted using TriReagent according to the manufacturer’s instructions (Astral Scientific Pty. Ltd., Caringbah, Australia; see also [36]). Several hundred individuals of representative developmental stages of all three species were fixed for *in situ* hybridization experiments as previously described [36, 37]. Specimens were either stored in 75 % EtOH or in 100 % Methanol at −20 °C.

### RNAseq and transcriptome assembly

Pooled total RNA of all above-mentioned developmental stages was used for the preparation of amplified short insert cDNA libraries (150–250 bp insert size) for *Nucula tumidula* and *Acanthochitona crinita* (Eurofins, Ebersberg, Germany). Both libraries (Kit version TruSeq SBS Kit v3) were sequenced together with another two bar-coded libraries in 1 channel of HiSeq 2000 with Illumina chemistry v3.0. Sequences were demultiplexed according to the 6 bp index code with 0 mismatch allowed. In both cases a PhiX library was added before sequencing to estimate the error rate of the sequences. Sequencing resulted in a total amount of 8.160 Mbp for *N. tumidula* and 7.147 Mbp for *A. crinita*. Paired-end reads with an average read length of 100 bp were obtained. These reads were subsequently filtered (rRNA removal) and adapter and low quality sequences were trimmed, normalized, and assembled *de novo* into contigs with the assembler Trinity [38]. The transcriptomes of *A. crinita* and *N. tumidula* comprised 166,556 contigs and 224,633 contigs, respectively. The sequencing strategy for developmental stages of *I. notoides* was described previously [36]. Briefly, RNA of developmental stages was sequenced by 454 technology by Eurofins (Ebersberg, Germany). After filtering, adapter and low quality read trimming, the reads were normalized and assembled *de novo* by Eurofins.

### Alignment and phylogenetic analysis

Amino acid sequences of bilaterian *Pax2/5/8* orthologs retrieved from NCBI were used in blastp searches against the assembled transcriptomes of *A. crinita*, *N. tumidula*, and *I. notoides*. The amino acid sequences with the highest sequence similarities of these blastp hits were aligned in ClustalX 2.1 (for accession numbers see Table 1) and edited manually in Geneious Pro 5.5.6 (Biomatters, Auckland, New Zealand, www.geneious.com). One alignment including only metazoan *Pax2/5/8* orthologs was performed in order to highlight the *Pax2/5/8*-specific domains (Fig. 2b), while another alignment comprising amino acid sequences of various Pax genes was done to show that *Acr-Pax2/5/8, Ntu-Pax2/5/8*, and *Ino-Pax2/5/8* cluster with their metazoan orthologs (Fig. 2a). The conserved motifs of the paired domains of the latter alignment were used to construct a maximum-likelihood consensus tree (100 bootstrap replicates) with the program Phylip v.3.695 [39].

**Table 1:** GenBank accession numbers of genes used for the phylogenetic analysis

Species name	Phylum	Gene name (Abbreviation)	Accession number
*Coeloplana willeyi*	Ctenophora	*Pax A*	BAF56224.1
		*Pax B*	BAF56225.1
*Clathria prolifera*	Porifera	*Pax2/5/8*	BAI66187.1
*Ephydatia fluviatilis*		*Pax2/5/8*	BAA36346.1
*Hydra littoralis*	Cnidaria	*PaxB*	AAB58291.1
*Hydra vulgaris*		*Pax2/5/8*	BAA36345.2
*Strongylocentrotus purpuratus*	Echinodermata	*Pax2*	XP_781786.3
		*Pax9*	XP_800234.2
*Saccoglossus kowalevskii*	Hemichordata	*Pax2A-like*	XP_006821852.1
*Ciona intestinalis*	Chordata	*Pax2/5/8*	NP_001027652.1
*Mus musculus*		*Pax1*	AAK01146.1
		*Pax2*	NP_035167.4
		*Pax3*	NP_032807.3
		*Pax5*	NP_032808.1
		*Pax7*	NP_035169.1
		*Pax8*	Q00288.3
*Homo sapiens*		*Pax2*	AAC63385.1
		*Pax5*	NP_001267479.1
		*Pax8*	Q06710.2
*Xenopus laevis*		*Pax2*	NP_001081941.1
		*Pax5*	NP_001079237.1
		*Pax8*	NP_001081941.1
*Danio rerio*		*Pax2*	AAD19287.1
		*Pax5*	NP_571713.1
		*Pax8*	XP_009298044.1
*Anolis carolensis*		*Pax5*	XP_008112815.1
*Trichuris trichiura*	Nematoda	*Pax2*	CDW54509.1
*Trichinella spiralis*		*Pax2*	EFV60539.1
*Acanthochitona crinita*	Mollusca	*Pax2/5/8*	KT380897
*Nucula tumidula*		*Pax2/5/8*	KT380898
*Crassostrea gigas*		*Pax2A*	EKC36239.1
*Lottia gigantea*		*Pax Beta*	DAA12512.1
*Idiosepius notoides*		*Pax2/5/8*	KT380899
*Platynereis dumerilii*	Annelida	*Pax2/5/8*	AGC12568.1
*Capitella teleta*		*Pox Neuro*	ELU04773.1
		*Pax3/7*	ABC68267.1
*Helobdella austinensis*		*Pax Beta1*	ABQ45870.1
*Euperipatoides rowelli*	Onychophora	*Pax2/5/8*	AJG44467.1
		*Pox Neuro*	AJG44468.1
		*Pax Alpha*	AJG44471.1
		*Pox Meso*	AJG44466.1
		*Pax3/7*	AJG44469.1
		*Pax6*	AJG44470.1
*Tribolium castaneum*	Arthropoda	*Shaven (Sv)*	EFA01334.1
*Drosophila melanogaster*		*Shaven (Sv)*	NP_524633.3
*Drosophila melanogaster*		*Paired (Prd)*	NP_723721.1
*Drosophila melanogaster*		*Eyeless (Ey)*	NP_524628.2
*Drosophila melanogaster*		*Pox Neuro*	NP_476686.1
*Drosophila melanogaster*		*Pox Meso*	NP_001036687.1
*Stegodyphus mimosarum*		*Pax2*	KFM62957.1
*Microplitis demolitor*		*Pax8*	XP_008547966.1

### Molecular isolation of *Pax2/5/8* sequence orthologs

RNA pooled from different developmental stages of *A. crinita*, *N. tumidula*, and *I. notoides*, respectively, was used for first-strand cDNA synthesis by reverse transcription using the First strand cDNA Synthesis Kit for rt-PCR (Roche Diagnostics GmbH, Mannheim, Germany). Gene-specific primers were designed from identified *Pax2/5/8* orthologs of *A. crinita*, *N. tumidula*, and *I. notoides* and transcripts were amplified via standard PCR. PCR products were size-fractioned by gel electrophoresis and gel bands of the expected length were excised and cleaned up using a QIAquick Gel Extraction Kit (Qiagen). Subsequently, cleaned-up products were cloned by insertion into pGEM-T Easy Vectors (Promega, Mannheim, Germany). Plasmid minipreps were grown overnight, cleaned-up, and sent for sequencing. All *Pax2/5/8*-gene orthologs were identified using the BLASTx algorithm screening the database of the National Center for Biotechnology Information (NCBI). All sequences and phylogenetic data that have been obtained in this study have been deposited in appropriate data bases.

### Probe syntheses and whole-mount *in situ* hybridization

From the miniprepped plasmids the probe template was amplified via standard PCR using M13 forward and reverse primers, and *in vitro* transcription reactions were performed with these templates, digoxigenin-UTP (DIG RNA Labeling Kit, Roche Diagnostics GmbH), and SP6/ T7 polymerase (Roche Diagnostics GmbH) for the syntheses of antisense riboprobes, according to the manufacturer’s instructions. Whole-mount *in situ* hybridization experiments were carried out as described previously [36, 37]. Briefly, specimens were rehydrated into PBT (PBS + 0.1 % Tween-20). They were treated with Proteinase-K (50-60 μg/ml for *Acanthochitona crinita*, 10 μg/ml for *Nucula tumidula*, and 25 μg/ml for *Idiosepius notoides*) in PBT at 37 °C for 15 min and prehybridized in hybridization buffer for 4 h or overnight at 65 °C for *A. crinita* and *I. notoides*, and at 56 °C for *N. tumidula*. Hybridization with a probe concentration of 0.5 to 1 μg/ml was carried out overnight at the same temperatures as those for prehybridization. For *I. notoides*, a minimum of 20 individuals per stage was investigated and for *A. crinita* and *N. tumidula* approximately 50 individuals per stage. In addition, negative controls were carried out with sense probes for all genes and developmental stages. The majority of whole-mount preparations were cleared in a 3:1 solution of benzyl-benzoate and benzyl alcohol, mounted on objective slides, and analyzed. The results were documented with an Olympus BX53 microscope (Olympus, Hamburg, Germany) and polyplacophoran and bivalve developmental stages were additionally scanned with a Leica confocal SP5 II microscope (Leica Microsystems,Wetzlar, Germany) using bright-field and autofluorescence scans as well as the reflection mode [40]. If necessary, images were processed with Adobe Photoshop 9.0.2 software (Adobe Systems, San Jose, USA) to adjust contrast and brightness. Sketch drawings were done with Adobe Illustrator CS5 software (Adobe Systems).

### Immunochemistry

Specimens stored in 75 % EtOH or 100 % MetOH at −20 °C were rehydrated and washed several times in PBS. Samples were rinsed thrice for 20 min each in PBS with 2 % Triton X-100 (PBT) to increase tissue permeability and were then blocked for 4 h in PBT with 3 % normal swine serum (NSS; Jackson ImmunoResearch, West Grove, USA) at RT. Specimens were incubated for 12 to 24 h at RT in a cocktail of primary antibodies directed against serotonin (raised in rabbit, polyclonal, Immunostar, Hudson, USA) or FMRFamide-related peptides (raised in rabbit, polyclonal; Biotrend, Cologne, Germany), and a primary antibody against acetylated α-tubulin (raised in mouse, monoclonal; Sigma Aldrich), all diluted 1:800 in PBT with 2 % NGS. After rinsing specimens five times for 20 min each in PBS at RT, secondary fluorochrome–coupled antibodies were applied in a 1:1.000 dilution over night at RT. These cocktails contained PBT with 1 % NGS and Alexa Fluor 488 (anti-rabbit; Invitrogen) and Alexa Fluor 633 (anti-mouse, Invitrogen), as well as 1 % Hoechst 33342 ((2'-[4-ethoxyphenyl]-5-[4-methyl-1-piperazinyl]-2,5'-bi-1H-benzimidazole trihydrochloride trihydrate); Thermo Scientific) for cell nuclei staining. Samples were rinsed in PBS five times for 20 min each at RT and subsequently mounted on glass slides in Fluoromount G (Southern Biotech, Birmingham, Alabama, USA). Glass slides were stored in the dark at 4 °C until the mounting medium had hardened. Specimens were investigated and results analyzed as described above. Isosurfaces of FMRFamide-like reactive and serotonin-like immunoreactive elements were created by surface rendering algorithms using the 3D reconstruction imaging software IMARIS (Bitplane, Zurich, Switzerland). Specificity of the FMRFamide-like and serotonin-like antibodies was tested by omission of the primary antibodies and rendered no signal.

### Statement of ethical approval

Animals were collected, anesthetized, and fixed according to internationally recognized standards (University of Queensland Animal Welfare Permit No. 158/09 “The cultivation of *Idiosepius* (pygmy squid) for studies in developmental biology” to BMD). Field work permission was granted by the ethics committee for every collection site of *I. notoides* used in this study.
